# Cellular immunity in COVID-19 and other infections in Common variable immunodeficiency

**DOI:** 10.3389/fimmu.2023.1124279

**Published:** 2023-04-26

**Authors:** Ragnhild Øye Løken, Børre Fevang

**Affiliations:** ^1^ Section of Clinical Immunology and Infectious Diseases, Division of Surgery, Inflammatory Medicine and Transplantation, Oslo University Hospital, Oslo, Norway; ^2^ Centre for Rare Disorders, Division of Paediatric and Adolescent Medicine, Oslo University Hospital, Oslo, Norway

**Keywords:** Common variable immunodeficiency (CVID), COVID-19, vaccine, opportunistic infection (OI), hypogammaglobulinemia

## Abstract

COVID-19 has shed light on the role of cellular immunity in the absence of humoral response in different patient groups. Common variable immunodeficiency (CVID) is characterized by impaired humoral immunity but also an underlying T-cell dysregulation. The impact of T-cell dysregulation on cellular immunity in CVID is not clear, and this review summarizes available literature on cellular immunity in CVID with a particular focus on COVID-19. Overall mortality of COVID-19 in CVID is difficult to assess, but seems not significantly elevated, and risk factors for severe disease mirrors that of the general population, including lymphopenia. Most CVID patients have a significant T-cell response to COVID-19 disease with possible cross-reactivity to endemic coronaviruses. Several studies find a significant but impaired cellular response to basal COVID-19 mRNA vaccination that is independent of an antibody response. CVID patients with infection only have better cellular responses to vaccine in one study, but there is no clear association to T-cell dysregulation. Cellular response wane over time but responds to a third booster dose of vaccine. Opportunistic infection as a sign of impaired cellular immunity in CVID is rare but is related to the definition of the disease. CVID patients have a cellular response to influenza vaccine that in most studies is comparable to healthy controls, and annual vaccination against seasonal influenza should be recommended. More research is required to clarify the effect of vaccines in CVID with the most immediate issue being when to booster the COVID-19 vaccine.

## Introduction

Studies of COVID-19 have shed light on the role of cellular immunity in the absence of humoral response in otherwise healthy patients and in patients with inborn errors of immunity (IEI) (1–6). Common variable immunodeficiency (CVID) is characterized by impaired humoral immunity and is the most common symptomatic IEI with a prevalence of 1:25 000 in adults (7). CVID is characterized by low IgG, IgA +/- IgM and either a poor serologic response to vaccines or low levels of class-switched memory B-cells, with the absence of profound T-cell deficiency or other causes of hypogammaglobinemia, as defined by the European Society for Immunodeficiencies (8). Patients typically present with recurrent airway infections with encapsulated bacteria, but are also prone to other infections and autoimmune and inflammatory complications (9, 10). A monogenic cause of disease is found in 10-20% of patients (11). CVID patients are also characterized by an inverted CD4/8 T-cell ratio, a relative CD4+ T-cell lymphopenia and markers of T-cell activation and exhaustion, pointing to an underlying T-cell dysregulation (12–16). The impact of T-cell dysregulation on cellular immunity in CVID is not clear, and we will here summarize available literature on cellular immunity in CVID with a particular focus on COVID-19.

## Cellular immunity in natural infections in CVID

### COVID-19

#### Mortality and morbidity

The course of COVID-19 in patients with IEI and CVID has been reported in several studies (17–27). Study designs vary and overall mortality of COVID-19 in CVID is difficult to assess, but a recent review by Tangye et al. estimated the case fatality rate in CVID to 7% as compared to 1-4% in the general population (28). Notably, known risk factors for severe COVID-19 as age, obesity and chronic lung disease are of significant importance also in CVID (19, 22–24). Lymphopenia is another known risk factor for severe COVID-19 in the general population and has been associated with a worse outcome also in CVID (19, 24, 29). Patients with Goods syndrome and untreated Severe Combined Immunodeficiency (SCID) seem particularly vulnerable to COVID-19 suggesting a particularly important role for T-cells when the humoral immune response fails. On the other hand, patients with pure B-cell deficiency in the form of X-linked agammaglobulinemia (XLA) have a risk of severe Covid-19 comparable to CVID (28, 30, 31). Clinical data thus present conflicting evidence regarding the potential role of cellular immunity in controlling COVID-19 in CVID.

#### T-cell responses

The role of T-cells in COVID-19 in CVID patients has been studied in several articles (1, 3, 5, 6). Kinoshita et al. report findings in five unvaccinated COVID-19 patients with primary antibody deficiencies (PAD) (6). Three of these patients had CVID with mild COVID-19 and were sampled 30-80 days after a positive SARS-CoV-2 test. The patients had CD4+ T-cell responses similar to healthy COVID-19 convalescents as assessed by cytokine production in spike, membrane and nucleocapsid protein stimulated peripheral blood mononuclear cells (PBMC) cultures.

The Hanitsch group has presented their work on T-cell responses to COVID-19 in CVID in two articles. In the first one, analyzing activation markers and cytokine production in stimulated PBMC cultures, Steiner et al. show that of 11 uninfected CVID-patients with no lymphopenia, seven had reactive CD4+ T-cells against SARS-CoV-2 spike proteins but none against nucleocapsid protein (3). Notably, four of these seven patients also had reactive CD4+ T-cells against spike protein from endemic coronaviruses. Healthy controls, both exposed and unexposed to COVID-19, had a higher frequency of all these reactive T-cell populations. The authors found a significant correlation between reactivity to SARS-CoV-2 and endemic coronaviruses in unexposed healthy controls, suggesting the possibility of cross-reactive T-cells.

In a second paper, Steiner et al. demonstrate strong T-cell responses in three unvaccinated CVID-patients with severe COVID-19 (1). The CVID patients were sampled 24-40 days after the debut of symptoms. All CVID-patients had CD4+ T-cells reactive to spike and nucleocapsid protein. Notably, frequencies of reactive T-cells were significantly higher in CVID-patients than convalescent healthy controls, but these controls had mild disease. Similar to their previous report, reactive CD4+ T-cells produced cytokines IFN-γ, TNF and IL-2 but here the strongest response was seen in CVID-patients. None of these CVID-patients with severe COVID-19 showed a serologic response. The relationship between serologic^nbsp;and cellular responses has also been studied in a different publication from the same group (2). In a group of immunocompetent convalescents of mild COVID-19 infection, CD4+ and CD8+ T-cells reactive to spike and nucleocapsid proteins of SARS-CoV-2 were found both in seropositive and seronegative patients. While similar reactivity also was found in healthy unexposed controls there was a significantly increased cytokine production in both convalescent groups. The authors conclude that there is evidence for specific T-cell responses in COVID-19.

### Other infections

CVID patients typically have increased frequency of respiratory tract infections with encapsulated bacteria like *Streptococcus pneumonia* and *Haemophilus influenzae* but also respiratory viruses (32–34). Less frequently, patients may present with gastrointestinal infections with pathogens like *Giardia lamblia*, *Campylobacter* or *Salmonella species* (32, 33). Clinically, impaired cellular immunity in CVID could particularly manifest itself through increased susceptibility to infections with opportunistic microbes like Cytomegalovirus (CMV) or *Pneumocystis jirovecci* (35, 36). These opportunistic infections tend to be a rare manifestation of CVID, and are associated with T-cell defects. The clinical consequences of T-cell dysregulation could include an altered immune response also to other pathogens.

#### Opportunistic infections overall

In a study by Oksenhendler et al. of infections in 252 patients with CVID, 16 (6%) patients presented with opportunistic infections like CMV or Pneumocystis jirovecci pneumonia (PCP) but we do not know the CD4 levels of the afflicted patients (33). In a seminal paper from the same group, Malphettes et al, reported that 28 of 313 CVID patients presented with opportunistic infections or CD4 <200 and termed these patients late combined immunodeficiency (LOCID) (36). A similar finding has been done by Cunningham-Rundles et al. where 5% out of 248 patients studied with CVID had CD4 cell counts lower than 200×10^6^ cells/L (32). Five of these lymphopenic patients presented with opportunistic infections, and 55 patients in the same study had mild or moderate infections that also pointed towards a T cell defect. Resnick et al. reported a higher number of opportunistic infections (15,4%) in a study of 473 CVID patients followed over four decades, but lymphopenic patients could have been included in this cohort (37).

#### CMV

CMV related disease occurs almost exclusively in patients with impaired cellular immunity. Kralickova et al. studied 32 patients to determine the frequency of CMV-related disease in CVID patients (38). Symptomatic CMV infection was documented in three CVID patients, where two patients were diagnosed with CMV pneumonia, and two patients suffered from CMV enteritis (one patient had both). None of these patients had severe abnormalities in T-cell subpopulations and all had CD4 ≥200 at the time of CVID diagnosis. However, all three patients had various other comorbidities, and these findings are in line with the studies by Cunningham-Rundles et al. and Resnick et al.

## Cellular response to vaccinations in CVID

### SARS-CoV-2 vaccination

Patients with CVID have been included in several studies on humoral and cellular responses to SARS-CoV-2 vaccination (**Table 1**) (39–56). Most studies assess the effect of two-dose basal mRNA vaccination, while some also include viral-vector vaccines and a booster dose. Cellular responses have been evaluated using different methods: (i) interferon-gamma release assays (IGRA), (ii) cytokine production assessed by enzyme-linked immunosorbent spot (ELISPOT) or flowcytometry, (iii) quantification of extracellular activation markers using flowcytometry or (iv) proliferation assays. In some studies, two methods have been used.

**Table 1:** Studies assessing cellular responses to SARS-CoV2 vaccination in CVID.

Study	n	Vaccine		Sample Week after 1st	Assay		Cellular response (Responders/total)		Comments
		Type	Doses		Stimulant	Read-out	CVID	HC	
Hagin (47)	13	BNT162b2	2	5	Spike protein (Miltenyi)	IL-2/IFN-γ ELISPOT (Diaclone)	9/13	11/11	Humoral response in all cellular non-responders
Salinas (40)	26	BNT162b2	2	4	Spike protein (JPT)	IFN-γ ELISPOT (Mabtech)	18/26	Continuous data	Reduced cellular/ humoral response in CVID
Arroyo- Sanches (42)	18	BNT162b2 (11), mRNA1273 (6), ChAdOx (1)	2	7-8	Spike protein (Mabtech)	IFN-γ ELISPOT (Mabtech)	15/18	49/50	Humoral response in 2/3 cellular non-responders. Increased humoral and cellular response in “Infection only” phenotype
Pulvirenti (53)	9	BNT162b2	2	4	Spike protein (JPT)	IFN-γ ELISPOT (Mabtech)	1/9	Continuous data	Reduced cellular response in CVID
Bergman (44)	11	BNT162b2	2	5	Spike protein (PE)	IFN-γ ELISPOT (Mabtech)	4/11	20/35	Median response similar in CVID and controls
						CD69+CD154+ CD4+ T-cells	9/14	44/44	Median response lower in CVID
Amodio (48)	14	BNT162b2	2	4	Spike protein (Miltenyi)	CD154+ CD4+ T-cells	10/14	17/18	No difference in T-cell subsets cellular responders vs nonresponders. Humoral response in 3/4 cellular non-responders
Sauerwein (46)	31	BNT162b2	2	9	Spike protein (Miltenyi)	CD25+CD134+ T_FH_ and T_Mem_-cells	Continuous data	Continuous data	Median response lower in CVID
Milota (52)	12	BNT162b2	2	8/26	Spike/ nucleo- protein (JPT)	IL-2/IFN-γ/TNF CD4+ T-cells	6/13 (w8) 6/12 (w26)	8/11(w8) 9/15 (w26)	Same proportion in CVID and controls
Shin (50)	8	mRNA-1273 BNT162b2	2	7-8	Spike protein (Miltenyi)	CD134+CD137+ CD4+ T_EM_	Continuous data	4/4	No cellular response in CVID after 1^st^ dose, similar response as controls after 2^nd^ dose
van Leeuwen (49)	56	mRNA-1273	2	8	Spike protein (Miltenyi)	IGRA (Qiagen, Euroimmun)	37/55	59/67	Limited correlation humoral/ cellular response. Cell non-resp compl phenotype
Kralickova (51)	34	mRNA-1273	2	6/16	Spike protein (Qiagen)	IGRA CD4+ (Qiagen)	14/34 (w6) 7/14 (w16)	No controls	Cellular and humoral response in 1/7 patients previously treated with rituximab
						IGRA CD4+CD8+ (Qiagen)	17/34 (w6) 7/17 (w16)	No controls	
Pulvirenti (54)	47	BNT162b2	3	w2 3rd	Spike protein (JPT)	IFN-γ/TNF CD4+ T-cells	17/47(TNF) 13/47 (IFN)	7/7(TNF) 7/7 (IFN)	No response in CD8+ T-cells in healthy controls or patients
Ainsua- Enrich (55)	12	mRNA-1273	3	28 (w4 after 3^rd^)	Spike protein (Miltenyi)	IFN-g ELISPOT (Mabtech)	8/12 (w8) 4/12 (w24) 7/11 (w28)	10/10 (all samplings)	Activation of CD8+ T-cells in 11/12 patients but magnitude lower than healthy controls.
						CD69+CD154+ CD4+ T-cells (also other)	8/12 (w8) 9/12 (w24) 9/11 (w28)	10/10 (all samplings)	

HC, healthy controls.

#### Defects in cytokine production

The first study presenting data on vaccine efficacy in IEI was authored by Hagin et al. using a spike protein stimulated IL-2/IFN-γ ELISPOT assay to evaluate cellular responses at week five after start of vaccination (47). In a group of 26 patients with IEI that included 13 patients with CVID, 19 patients had a positive cellular response similar to healthy controls. Notably, all controls had a cellular response to vaccination whereas four of the patients with CVID were non-responders. These patients did have a humoral response to vaccination with anti-S antibody levels that were variable but blunted. Interestingly, patients with XLA had a stronger cellular response than both healthy controls and the rest of the IEI group.

In 41 patients with CVID that received basal mRNA vaccination, Salinas et al. found no increase in spike protein induced IFN-γ producing cells in patients as assessed by an ELISPOT assay four weeks after the start of vaccination, and found defective cellular response in 30% of patients (40). In contrast, all six XLA-patients did respond. The CVID-patients again had a significant but blunted humoral response to vaccination as compared to healthy controls.

Antoli et al. demonstrated an impaired cellular response to basal vaccination in a group of 28 CVID patients (27 mRNA, 1 viral vector vaccine) sampled four weeks after the second dose (43). Patients had significantly lower IFN-γ levels in a SARS-CoV-2 specific IGRA-test compared to healthy controls, and 8 patients had no response. The authors identified several predictors for poor humoral responses, including an inverted CD4/CD8 ratio, but these predictors were not associated with an impaired cellular response.

In a study looking at the effect of basal vaccination in 18 patients with CVID (17 mRNA, 1 viral vector vaccine), Arroyo-Sánchez et al. report 83% positive response four weeks after the second dose using a spike protein stimulated IFN-g ELISPOT assay (42). Again, this was significantly lower than the 98% positive cellular response observed among healthy controls, and likewise 15 of 18 CVID-patients were anti-S1 IgG positive compared to 50 of 50 healthy controls. Three patients had no cellular response to vaccination but two of them did mount an antibody response. Notably, CVID patients with an infection only phenotype seemed to have a better humoral and cellular response to basal COVID-19 vaccination than patients with inflammatory complications.

Bergman and colleagues present their findings of COVID-19 vaccine responses in a large cohort of patients with IEI in two articles (41, 44). In their second article they show that in 11 patients with CVID and a group of healthy controls, there was a significant and comparable cellular response at week five after the start of basal vaccination as assessed by an IFN-γ ELISPOT assay. There was also an increase in spike protein activated CD4+CD69+CD154+ T-cells in CVID-patients and controls after vaccination, and here response was impaired in patients as compared to controls. The antibody response in CVID was also impaired with anti-spike IgG detected in 68 vs 100% of patients and controls, respectively. Interestingly, they found that low CD4+ T-cells and high CD21^Low^ B-cells were associated with a poor humoral response to vaccines, but there were no data on any relationship to the cellular response.

In a large cohort of over 500 patients with IEI that included 212 patients with CVID, van Leeuwen et al. looked at the effect of basal vaccination 8 weeks after the start of vaccination (49). Cellular responses were evaluated in a subgroup of the cohort (56 CVID patients) using spike protein IGRA and finding a significantly reduced proportion of responders among CVID patients (67%) versus healthy controls (88%). Notably, while CVID patients had impaired cellular responses compared to healthy controls this was not found in other groups of IEI like XLA and IgG specific antibody deficiency. Seroconversion among CVID patients were significantly reduced compared to healthy controls (81% vs 100%, patients and controls, respectively).

Looking at the long-term effect of basal vaccination, Kralickova et al. sampled a group of 46 patients with IEI that included 34 patients with CVID 6 and 16 weeks after the start of vaccination (51). Cellular responses were analyzed using spike protein IGRA-assays that stimulated CD4+ and CD4+/CD8+ T-cells, respectively. A positive CD4+ T-cell response was noted in 41% and 50% of CVID patients at week 6 and 16, respectively, while the combined CD4+/CD8+ assay showed a negative trend over time with 50% and 41% positive at week 6 and 16, respectively. Seven CVID patients had previously been treated with rituximab and notably only two of these patients were able to mount a cellular response at week six.

Milota et al. have also looked at the long-term cellular effect of basal vaccination in CVID following a group of 12 CVID patients over 6 months (52). Cellular responses were evaluated by flowcytometry of intracellular IL-2/IFN-γ/TNF in spike/nucleoprotein stimulated PBMCs. CVID patients had a similar proportion of spike protein reactive CD4+ T-cells as controls at one and six months after vaccination (46 vs 73%, and 50% vs 60%, respectively). Notably, anti-receptor binding domain (RBD) antibody were significantly and incrementally lower in CVID patients compared to controls at 1, 3 and 6 months.

In a study that included 9 CVID basal vaccine recipients and 15 CVID COVID-19 convalescents, Pulvirenti et al. showed that both groups had impaired T-cell responses as assessed by spike protein IGRA compared to healthy controls (53). Immunized patients were sampled at week 4 after the start of vaccination whereas convalescents were sampled at week 12 after first positive SARS-CoV-2 PCR. The same group further explored the role of T-cells in a different study that included 47 CVID patients with at third (booster) vaccine dose (54). Patients were sampled two weeks after the third dose and cellular response were measured in spike protein stimulated cultures of PBMC by staining for IFN-γ, TNF and CD40L. Notably, the spike protein induced expression of CD40L and TNF in CD4+ T-cells were significantly reduced in CVID patients compared to controls, while the expression of IFN-γ was similar. Likewise, even if CVID patients had increased levels of anti-S IgG after the third dose, they were still significantly lower than what was seen in healthy controls.

Ainsua-Enrich et al. report findings in a study that included 12 CVID-patients receiving three mRNA vaccine doses (55). All healthy controls had positive spike protein IFN-γ ELISPOT at week 8 and 24 after basal vaccination, while this was found in 67% and 33% of patients with CVID, respectively. After receiving a third mRNA vaccine dose, the proportion of positive CVID patients increased to approximately 70%. CVID-patients also had a significant but still impaired response in activated CD8+ T-cells compared to controls at week 8 and 28.

#### Defects in activation markers

In an early study of 21 patients with IEI that included 14 CVID-patients, Amodio et al. found a significant increase in spike protein activated CD4+CD40L+ T-cells at week four after the start of basal vaccination (48). This increase was significantly lower than what was seen in healthy controls, and four of the 14 CVID patients did not respond. There were no significant differences in pre-vaccination T-cell subsets between cellular responders and non-responders in the CVID group. Three of the four cellular non-responders had a serologic response comparable to healthy controls.

As noted, Bergman and colleagues found an increase in spike protein activated CD4+CD69+CD154+ T-cells after basal vaccination in CVID-patients that was significantly lower than what was seen in healthy controls (41). In an in-depth article from the same vaccine study, Gao et al. present T-cell profiles of 48 patients with IEI, including CVID and XLA (56). Overall, they found that patients with IEI had a relatively high frequency of spike-specific polyfunctional CD4+ T-cells, while the frequency of spike-specific CD8+ T-cells were lower but comparable to healthy controls. Interestingly, they found that XLA patients had a strong cellular response despite their lack of humoral response, but similar data on CVID humoral non-responders are not presented.

Sauerwein et al. studied the response of CD4+ T-cells to basal mRNA vaccination in 31 CVID patients a median of 6 weeks after the second dose (46). Using a spike protein induced activation marker assay they found reduced activation of CD4+ memory T-cells and CD4+ circulating follicular T-cells in CVID patients vs healthy controls. Anti-spike IgG levels were also significantly lower in CVID patients compared to controls with 16 of 31 patients defined as non-responders. These non-responders were characterized by reduced proportion of CD4+ vaccine specific memory T-cells as compared to responders.

In an article on predictors of poor immune responses after basal mRNA vaccination, Shin et al. found that a group of 12 CVID patients had a delayed cellular response to vaccination as compared to healthy controls (50). The cellular response was evaluated using a spike protein induced activation marker assay identifying CD4+OX40 + 4-1BB+ effector memory T-cells.

#### Defects in proliferation

The above-mentioned study by Sauerwein et al. included a spike-protein induced 3H-thymidine proliferation assay. The assay showed significantly reduced proliferation in a group of 16 vaccinated CVID patients as compared to 14 healthy vaccinated controls (46).

#### Overall response to SARS-CoV-2 vaccination

**Figure 1:**
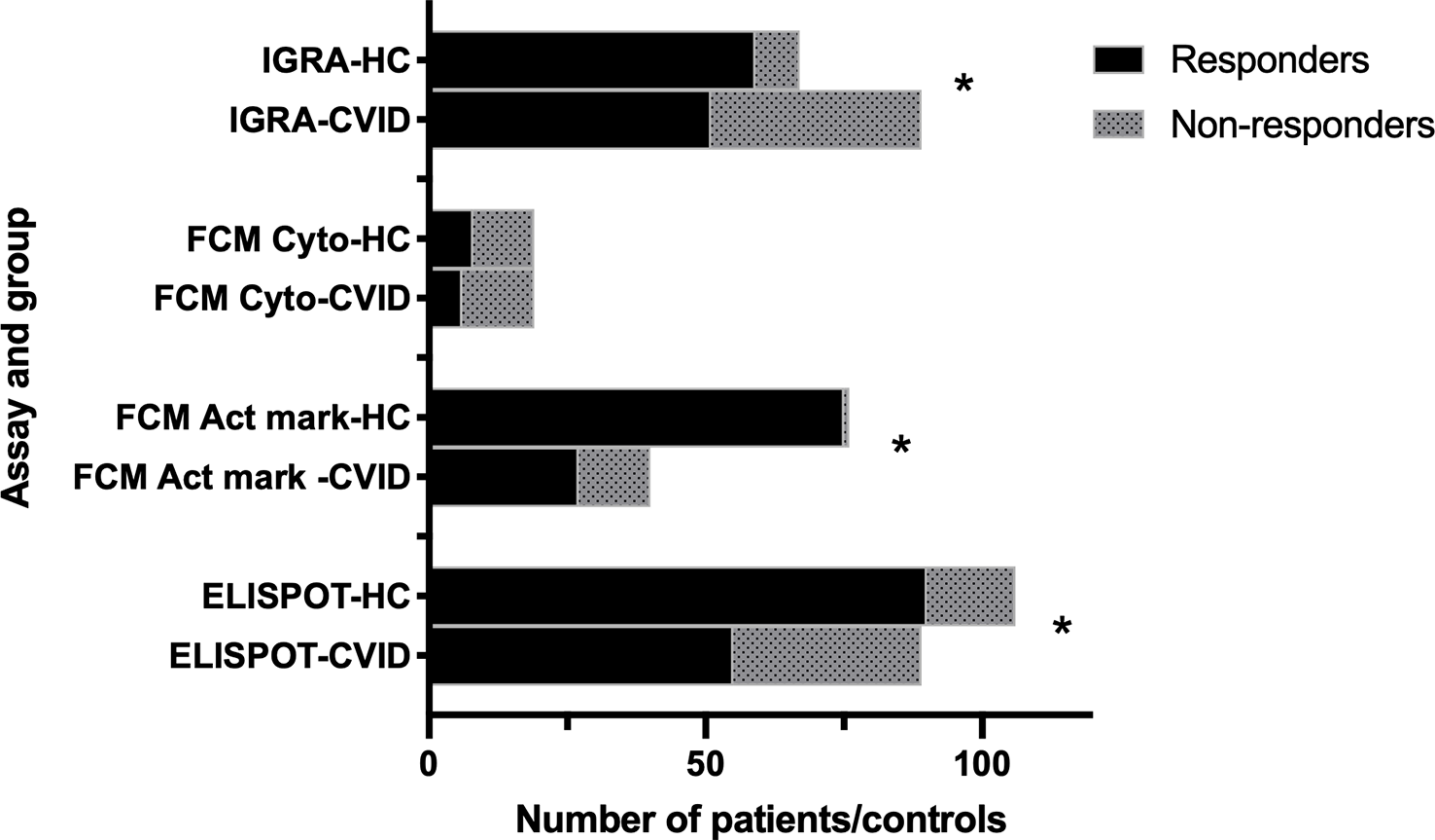
Cellular response to two doses of SARS-CoV-2 vaccination in CVID and healthy controls. Summary of data from studies using different assays (IGRA (49, 51); FCM Act mark (41, 48, 50, 55); FCM cyto (52); ELISPOT (40, 41, 42, 47, 53, 55). HC: healthy controls. FCM cyto: flowcytometry of cytokine expression. FCM Act mark: flowcytometry of activation markers. *p<0.001 as assessed by Chi-square test.

Summarizing the studies performed 4-8 weeks after the start of vaccination assays using ELISPOT, IGRA and flowcytometry of activation markers, all detect a significantly impaired response in CVID patients compared to healthy controls (**Figure 1**).

### Other vaccines

Patient with CVID have reduced antibody response to several vaccines, but notably, the determination of vaccine-specific T-cell responses that could serve as markers of cellular immunity in CVID is challenging and not routinely used.

#### Pneumococcal vaccine

CVID patients will typically have an impaired antibody response to vaccines containing T cell independent antigens like polysaccharides, but interestingly, pneumococcal vaccine induction of cytokines not related to B-cells are also found to be impaired (57–59). This is demonstrated in a study by Hong et al, where Pneumovax-23-induced secretion of IL-6 and TNF-alpha by monocytes was significantly lower in 14 patients with CVID as compared to controls (59).

#### Influenza vaccine

CVID patients have an impaired humoral response to influenza vaccine, while data on cellular responses are variable. In their study of 8 CVID patients, Hanitsch et al. found that the influenza-specific antibody and T-cell cytokine responses (vaccine specific IFN-γ, TNF-α and IL-2) in the CVID group were similar to that of the healthy controls (60). A similar finding is described in case reports from Pedersen et al, while Friedmann et al. found that cellular immunity is preserved in most CVID patients after vaccination with the seasonal flu vaccine (61, 62). In contrast, van Assen et al. demonstrated a reduction in vaccine specific cytokine-producing CD4^+^ T cells in 15 CVID patients compared to healthy controls (63).

## Discussion

**Figure 2:**
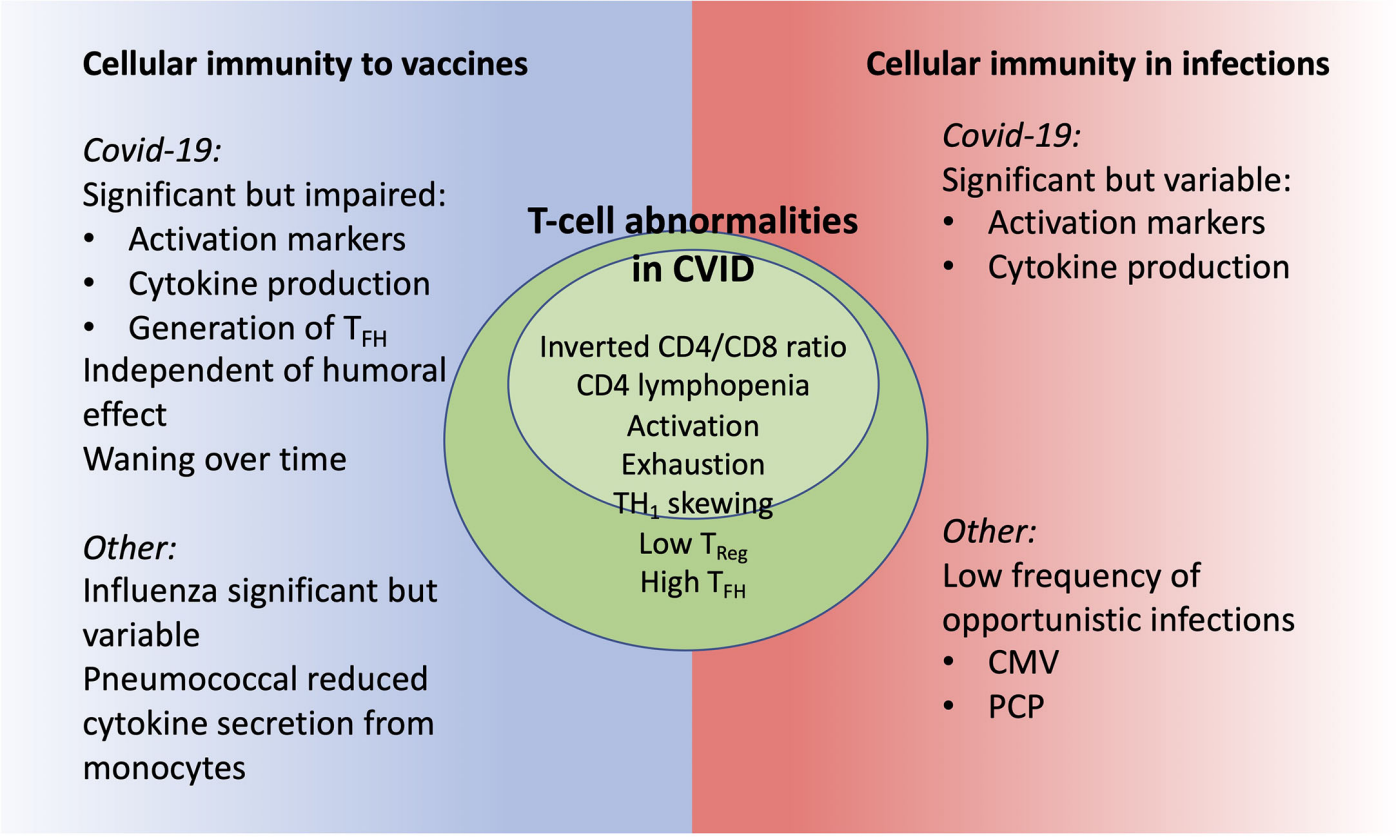
CVID patients have signs of T-cell dysregulation with potential impact on cellular immunity as seen in clinical and *in vitro* studies.

Patients with CVID have a variable immunological phenotype that is reflected also in the role and function of cellular immunity. Summarizing literature related to COVID-19 and other infections and vaccines, we find that most CVID patients have preserved clinical or *in vitro* cellular immunity with significant impairment in some patients (**Figure 2**). The definition of CVID and inclusion/exclusion of patients with T cell lymphopenia is of importance but notably, there is T-cell dysregulation also in CVID-patients with CD4 >200 x 10^9^/l (12, 16).

CVID patients with COVID-19 have a relatively good prognosis and so far, no certain association between clinical or immunological phenotypes and severity of COVID-19 has been found (18, 19, 21, 28). The dominating risk factor for severe COVID-19 in CVID mirrors that of the general population, including lymphopenia. Lymphopenia is a risk factor also for other infections in CVID, including opportunistic infections. Several large cohort studies of CVID have identified subgroups of patients with a history of CMV, PCP or other opportunistic infections associated to T-cell deficiency. Regardless the definition of CVID, any history of opportunistic infections should prompt increased awareness when using immunomodulating drugs and the consideration of anti-microbial prophylaxis.

The role of cellular immunity in CVID is of particular interest in assessing the effect of vaccination. Previous studies have shown that most CVID patients have a cellular response to influenza vaccine comparable to healthy controls, and a number of studies now show a significant but impaired cellular response to COVID-19 vaccines. The response is however not generally impaired, as many patients will have a strong response while some are non-responders. T-cell dysregulation is associated with a poor antibody response, but interestingly a similar association has not been found for the cellular response (41, 43). The study from Amodio et al. show no difference in pre-vaccine T-cell subsets between CVID cellular responders vs non-responders, but the studies of Arroyo-Sánchez et al. and van Leeuwen et al. suggest a better response in CVID patients with infection only (42, 48, 49). CVID-patients recently treated with rituximab have particularly poor cellular response to vaccination, and if possible, this treatment should be paused before vaccination (51).

Overall, as stated by van Leuween et al, the correlation between humoral and cellular response to vaccination seems limited (49). So, while serologic testing for COVID-19 antibodies is widely available, it is not a reliable guide to any cellular response. Testing for cellular vaccine response should therefore also be implemented in the clinic. The identification of humoral and cellular non-responders is important in assessing need for further follow up, including selecting patients for antiviral treatment and prophylactic or therapeutic SARS-CoV-2 monoclonal antibodies. There is a waning of both cellular and humoral response over time after basal vaccination, but the studies of Pulvirenti et al. and Ainsua-Enrich et al. show a positive response to a third dose (54, 55).

The clinical effect of vaccines is uncertain in CVID, but immunological data support recommending vaccines also in this patient group. In general, immunocompromised patients have been observed to be undervaccinated, and potential advantageous effects from vaccines may previously have been underplayed. With the exception of live vaccines, the vast majority of vaccines are safe and well tolerated in immunodeficiencies like CVID. Annual seasonal influenza vaccine should be recommended, while vaccinations against pneumococci, Haemophilus influenzae type B and meningococci should be considered, in particular for at-risk patients. The justification can be supported by the heterogeneity of CVID patients as a group and their varying level of responses.

The most effective way to vaccinate against most pathogens involves evoking both a humoral and cellular immune response, and research in the last years have shed light on T-cell-inducing vaccines against various pathogens (64, 65). This could be of particular importance for patients with an impaired humoral response as CVID. More research is required to clarify the effect of vaccines in CVID with the most immediate issue being when to booster the COVID-19 vaccine.

## Author contributions

BF conceptualized the study, reviewed articles and drafted the manuscript. RL reviewed articles and drafted the manuscript. All authors contributed to the article and approved the submitted version.
